# Abstracts and Gleanings

**Published:** 1886-08-20

**Authors:** 


### ABSTRACTS AND GLEANINGS.

**The Treatment of Acute Sporadic Dysenteries.**—Dr. Q. C. Smith thus writes in the *Southern Practitioner*, for July: The remedies we have found most useful in the treatment of acute dysenteries, are as follows: Belladonna, ipecac, capsicum, gelsemium, aconite, iodine, nux vomica, spirits turpentine, cinchona, baptisia, aromatic spirits amonia, salicin, lupulin, logwood, subnitrate of bismuth, and vegetable charcoal. However, no one case is likely to require the use of so many remedies as all those just enumerated; but 'tis rare that other remedies than those just mentioned are required to successfully treat even severe cases of acute dysentery. Sporadic acute dysentery is generally preceded by a diarrhœal condition of one or two or more days, and when first called to a severe case of acute dysentery, we usually find the patient in great pain, of a more or less paroxysmal character, which is greatly aggravated at each frequent alvine effort, small bloody mucus discharges, lower abdomen tender on pressure, renal secretion greatly diminished or suppressed, soft pulse, more or less fever, thirst, irritable stomach, foul tongue, and loss of appetite. Such cases are often aggravated and seriously complicated by the administration of opium previous to the physician being called, who may be so unwise as to continue the administration of this delusive, but most pernicious drug—in the treatment of dysenteries. To quickly relieve such a case as we have just briefly outlined, we would begin by immediately giving a hypodermic of 1-60 to 1-30 grain sulph. atropia, cover the abdomen with dry, thick, hot cloths, applying soothing liniment to the abdomen if there be great pain, frequent *small* drinks of cool, acidulated drinks, or plain water. Soon the patient will be greatly relieved of pain, and the stomach in retainable condition. Then, say an hour after the hypodermic, begin the administration of something like the following:

℞. Fl. ext. belladonna....................ʒ j,

Green tr. gelsemium....................ʒ ij,

Tr. aconite rad. (Fleming's)....................gtt. iv,

Arom. spirits ammonia....................℥ ss,

Elix. lactopeptine, q. s. ft....................℥ ij.

M. Ft. sol.

S.—Teaspoonful every one to three hours, as may be required to control pain in the bowels.

Also:

℞. Ex. cinchona,

Ex. lupulin, } aa....................gtt. xii,

Ext. capsicum....................gtt. ij,

Ext. ipecac....................gtt. iv.

M. Ft. 12 pills and silver coat.

S. One pill three times a day—preferably just after meals.

Should the renal secretion be suppressed, or notably diminished, spirits turpentine, prepared as follows, would be in order:

℞. Spirits turpentine, Sugar of milk, Pure sugar, } aa ........................ ʒ j,

Oil sassafras .................................... gtt. iv,

Chloroform water ................................ ℥ ss.

Mix well.

Add.

℞. Fl. ext. ipecac .................................... gtt ij,
Cinnamon water, q. s. ad ........................ f. ℥ ij,

M. Ft. emulsion.

S. Teaspoonful every two to four hours until renal secretion is restored.

Spirits turpentine is also a valuable remedy in the more advanced stages of many cases of dysentery. But should the case have gone on for several days, from bad to worse, then other remedies, in addition to those just formulated, are often beneficial.

If the alvine discharges are very fœtid, vegetable charcoal, spirits turpentine, and baptisia are in order. If there be a tendency to diarrhœa, then salicin, subnitrate of bismuth, and logwood would be appropriate. In malarial dysentery—in addition to the remedies formulated—cinchona, iodine, ipecac, and arsenic, would be specially indicated.

In all forms, and in all stages, special attention should be given toward aiding digestion, for the diet of dysenteric patients is a matter of the first importance in all cases. For with the horizontal rest, proper alimentation, and care, many cases will speedily recover without the use of drugs. But only one or two kinds of food should be given at any one meal, and food should not be taken oftener than three times a day.

The prepared infant foods, and pepsin, serve a valuable purpose in many cases. Well-cooked and seasoned green salads with vinegar, good ripe fruits, cooked or raw, such as peaches, apples, grapes, oranges, lemons, dates, and figs, farinaceous foods, soups, well broiled and seasoned fat mutton chops, and beefsteaks, good fresh sweet milk, and butter milk, with fresh butter on toasted light bread. Such are the articles of food we usually prescribe for dysenteric patients, with satisfaction to ourselves and benefit to the patient.

*Opium*, mercury, and starvation are responsible for thousands of preventable deaths from dysentery.—*Medical and Surgical Reporter.*

**The "Cent" Sign in Pleurisy.**—Professor Pitres, of Bordeaux, indicates a new sign in ausculation. Dr. Davezac describes it as follows, in the *Journal de Medicine de Bordeaux*. The patient is seated, and is ausculated in the dorsal region. An assistant places a cent on the thorax, in different parts according to directions, and percusses. The ear of the ausculator listens at the opposed corresponding parts. The healthy side is first examined; then the side with pleurisy, where the note is much higher. A clear metalic sound indicates pleuritic effusion ; when this sound is absent, there is no effusion.—*Quarterly Compendium.*

**Anti-Rheumatic Mixture.**—A. Fort, says L'Union Medicale, (St. Louis *Courier of Medicine*) has found the following a serviceable combination :

℞. Potass. iodidi..............................ʒ v,

Potass. bromidi,..............................℈ iv,

Gentianæ..............................℥ xviij,

Tr. iodinii..............................20 drops.

M. Sig. A tablespoonful morning and evening in chronic articular rheumatism. Besides this, paint the painful joints with tincture of iodine.

**Small Doses.**—The present tendency in prescribing is to elegance and pleasantness. Although we have capsules, wafers, sugar and chocolate coatings, yet the drug may prove inert by the insolubility of the coating. Since the discovery of various alkaloids, small doses have become more common. If drugs be effectual in small doses frequently repeated, why not prescribe small doses ?

But do not understand me to say that we can prescribe for all diseases in this manner. There are some troubles which are only overcome by heroic doses.

In diphtheria, scarlatina, follicular tonsillitis, potassium chlorate in 1-grain doses every half hour affords much relief, and is curative.

One-grain doses of croton chloral every half hour in many forms of neuralgia is beneficial.

In obstinate urticaria, salicylate of soda, in 2-grain doses every half hour acts well ; also drop-doses of balsam of copaiba every half hour.

The vomiting of drunkards is often helped by ½-drop doses of Fowler's solution every half hour. This is also good in vomiting of pregnancy.

In erysipelas, the muriate of pilocarpine, 1-10 grain, hypodermically.

Wine of ipecac in drop-doses every fifteen minutes will often arrest obstinate vomiting caused by cancer ; also useful in children.

For vomiting of infants, A. A. Smith, of New York, has used 1 grain of calomel to 1 ounce of lime-water ; to this add 1 pint of pure water, and give a teaspoonful of the mixture every ten minutes.

In wheezing and cough of children with bronchitis, good results may be obtained with tartar emetic, 1 grain to 2 pints of water ; teaspoonful every half hour.

Sick headache is often relieved by 1 drop of tincture of nux vomica every five minutes.

One of our best remedies for inflammation of the bladder is tincture of cantharides, 1 drop every hour.

In excessive menstruation, fluid extract of ergot has been successfully used in minim doses every half hour, for six or eight hours before the expected flow.

A simple febrile movement, with hot, dry skin, full and bounding pulse, may be relieved by $\frac{1}{2}$-drop doses of tincture of aconite root every half hour ; also useful in acute nasal catarrh.

Sub-acute nasal catarrh, with abundant secretions, is often allayed by minim doses of tincture of belladonna every half hour, until eight or ten minims are taken.

In malarial fever, when quinine fails, picric acid, grain $\frac{1}{8}$, in combination with ammonia, is used with benefit; also beneficial in pertussis.

In asthma, with indigestion and anæmia, Fowler's solution in 1-drop doses often proves remarkably beneficial.

Apomorphia, grain 1-200 three or four times a day, often produces brilliant results in spasmodic cough.

Cannabis indica, grain $\frac{1}{3}$–$\frac{1}{2}$, given for weeks, is a useful agent in the treatment of megrim.

Atropia, in doses of 1-200 of a grain usually controls night sweats.

Digitalis, in small doses frequently repeated, exerts a beneficial influence over different kinds of hemorrhages.

Many troubles could be treated with small doses, and benefited as much, and often more, than to administer larger doses.—*Nashville Journal of Medicine and Surgery.*

**Education and Care of the Idiot.**—To go back to the question so often asked: 1. Can anything be done for the idiot? The emphatic answer is, yes! 2. What can be done? This we cannot answer as quickly. It depends upon the grade of intellect of the child; that is, how far removed from the mental state of normal children. To begin with the higher grades of the feeble minded: we can do for them the same things as are done for the children in the public schools, only taking more time and stopping far short of the normal child's capacity. We expect to do no more than to teach the child to read; enough to read simple stories, newspapers, and the like; to write enough to read and write letters; a very little of local and general geography, and enough arithmetic to count and reckon money; also tell the time; to know the days, weeks, months, seasons, etc.; and perhaps a very slight knowledge of the physiology of their own bodies. This can only be done in the best cases; but in addition to this intellectual training we may be able to accomplish much more by industrial education; the child can be taught to work. If a girl, domestic work, sewing and some fancy work; and if a boy, domestic work, farm work, and many simple occupations in the shop, like making brooms, seating chairs, repairing shoes, making mats, etc.

These are the things for which "the world is to give them a (whole or a part of a) living for knowing or doing," and in many cases it can be done. But there are cases less fortunate, those who cannot learn as much, and without being able to read and write, intellectually, they only improve in so far as to understand what is said to them, without being able to articulate themselves; but industriously they can learn to do much in the way of domestic duties, chores, etc. If of a lower grade of intellect still, perhaps all that can be done will be to teach the child better behavior, to attend to his natural wants, to feed, dress, and bathe himself, and in many cases to amuse and occupy himself; in short, to do enough to make himself less a burden to his friends. In the case of the lowest grade, the drivelling idiot, even less can be done. There is no intellect to develop, and perhaps none is ever awakened, or at most only a very few symptoms of intelligence may be shown, and here the education of course can only be that of attention to his own person, to make known his natural wants, that he may be assisted, and in a measure to care for himself, also to induce a kind of instinctive or reflex habit or education. By this he becomes less an object of disgust and less a burden to his friends. —*Dr. A. G. Smith, in Boston Med. and Surg. Journal.*

**The "Dead-Finger Symptom" in Bright's Disease.**— In the *Giornale Internazionale delle Science Mediche*, No. 3, 1886, we read: This is a sensation similar to that experienced when the finger is immersed in snow, or exposed to a great degree of cold. The patients complain of formication, painful sensations, and sometimes the finger-tip becomes anæmic, white, and numb. This symptom is usually of very brief duration. In one patient it will last a few seconds only, but will reappear whenever the attempt is made to grasp any object; in another its duration will be for five or six minutes, and it will be noticed to recur at longer or shorter intervals, as one or two days or a week; finally, a third will recall its appearance on a single occasion only during the course of his disease, when it may last for a quarter of an hour. The symptom is localized now in one finger, now in another, the little finger being the one most frequently affected, the middle, the ring, the index finger, and the thumb coming next in order of frequency. The phenomenon may appear at the beginning of Bright's disease or near its termination, but is of greater diagnostic importance in the former case, since the other symptoms of the affection may at this time be insignificont or even absent. As to the pathogenesis of this sign, Dr. Soyer believes that it is the first degree of local asphyxia of the extremities, and regards it as allied to symmetrical gangrene as sometimes observed.—*Quarterly Compendium.*

**The Treatment of Compound Fractures by Wiring and Drainage.**—In the procedure I am about to describe, there is no single detail which is strictly original, but the procedure as a whole deserves the attention of the profession. When a comminuted fracture comes under my observasion, the affected part and its surroundings are carefully cleansed and shaven. Soft parts so ragged and contused, and so situated as to produce separation of the fragments and to act as foreign bodies; the external wound being enlarged, if necessary, for this purpose. If the part affected be a lower extremity, this is flexed at an obtuse angle. The fragments are then approximated in the most normal position possible.

For this purpose projecting sharp points, if denuded and acting as irritants, may be removed, but an effort should be made to retain all such fragments as a means of support. When the best possible approximation is secured, the fragments are wired together; silver wire (when of good quality) is preferable, but iron wire is the most attainable and can be readily disinfected by means of heat and rendered properly flexible. Drainage is secured by means of large tubes from the most dependent point. The whole extremity is then covered with an extensive thick antiseptic dressing and incased in a plaster cast, removable at each dressing. The present procedure has certain very demonstrable advantages.

1. All points of bone and injured tissue fragments, *likely* to act as irritants, are removed, except where bone point is needed for mechanical support.

2. Proper retention of the fragments in place is presumably secured, thus avoiding any possible danger of the fragments overriding and injuring the soft tissues.

3. There is no extension needed, which avoids the necessity of complicated apparatus and procedures such as too often interfere with proper antiseptic dressings.

4. The bones unite quicker, for reasons which will be obvious when the principles which underlie all procedures to secure union of ununited fractures are recollected.

5. As a rule, it will be apparent that there can be little if any shortening.

6. Drainage prevents inflammation by preventing the accumulation of fluids.—*Dr. W. P. Verity, in Jour. Amer. Med. Asso'n.*

**Tuberculosis.**—A striking illustration of the value of the bichloride of mercury in the treatment of tuberculosis may be found in the case of S. T. M——, aged thirty eight years, who came, October 23, 1885, in a very feeble and emaciated condition, suffering from severe dispnœa, hoarseness, frequent chills followed by high fever, and colliquative sweats. Examination showed extensive infiltration of the epiglottis and the walls of the larynx. The vocal cords were concealed behind the swollen tissues above. The cough and expectoration seldom ceased more than five minutes at a time during the entire day.

The sputum was so rich in tubercle bacilli that mounted preparations of it were used as samplas for illustration in teaching. This man got a spray of the bichloride of mercury, prepared as follows:

℞. Hydr. bichloridi ............................ gr. ij,
Aquæ destillatæ ............................ O i,
Sodii chloridi ............................ ʒ i.
M. Ft. solutio.

He was ordered pills of the bichloride gr. 1-40 each, one before each meal and at night, and a pill composed of asafœtida gr. iij, and extract of nux vomica gr. ¼, to be taken at the same time. In six weeks he was walking the fields five or six miles daily, hunting game. He was married last January, and is now out West.—*Progress.*

**Permanent Drainage in Ascites.**—Dr. Aug. C. Calle read a paper on this subject before the New York Academy of Medicine (*Medical News*, February 13, 1886.) Two cases were related in full: the first patient, an elderly man, suffering from cirrhosis of the liver, had already been tapped nine times. Instead of merely resorting to another tapping, Dr. Calle inserted a short drainage tube through an incision below the umbilicus, and applied antiseptic dressings as required. At first two or three pints of fluid were evacuated daily, but the quantity gradually diminished, and finally the discharge ceased altogether. The general œdema and other distressing symptoms disappeared entirely. No bad results followed the operation, except an eczema, due to the constant moisture, and readily overcome by protecting the skin. The patient was enabled to go about attending to his usual occupation for nine months, and entirely comfortable. He died finally of heart failure, the ascites and general anasarca not having returned. On post mortem, cirrhsosis of the liver was found, and there was no evidence of peritonitis at the point where the tube had been inserted.

The second case, also one of cirrhosis of the liver, and in a very bad condition before operation, was also completely relieved, and lived in comfort for a considerable time, dylng finally of heart failure. No autopsy was obtained.

Dr. Calle then discussed the symptoms most troublesome and dangerous in ascites; the collateral circulation in cirrhosis of the liver; the ways in which relief of abdominal pressure might be expected to act beneficially, and the best means of accomplishing this giving a number of suggestions as to technique, and recommending strict antiseptic precautions, though stating that the peritoneum under such circumstances is well known to be less susceptible to infection than in the normal condition—*American Journal Medical Sciences.*

**Case of Re-Injection of Blood during Amputation at the Hip-Joint, with Rapid Recovery.**—In a case of strumous disease affecting both hips, the left knee and the left elbow, with a large abscess connected with the left hip, the patient being in very feeble condition, amputation at the latter joint became necessary. The limb having been exsanguinated to the middle of the thigh, and a powerful elastic tourniquet applied at the groin, a rapid circular cut was made right down to the bone in the upper part of the thigh, the femur sawn through, the femoral artery and also some smaller vessels tied, and the tourniquet removed; some hemorrhage still occurring from a few small vessels, they were also litigatured. All the blood which escaped, both from the femoral artery and the smaller vessels, amounting to eleven ounces, was caught in a vessel containing a solution of phosphate of soda, and reinjected into the deep femoral vein. By an incision on the outer side of the thigh, the head of the femur was then dissected out. The wound was dressed antiseptically. The patient suffered no shock whatever, nor depression of temperature after the operation. For the first few days he was flushed and had a fuller pulse than before the operation, but he had no rise of temperature.

The weakness and anæmia of the patient, together with the increased vascularity of the parts due to the disease, rendered it very likely that he would not have survived the operation, had not the greater part of the blood lost been reinjected—the fact being that from the exsanguinfication of the leg, together with the re-infusion, there was probably an ultimate gain of blood after the operation.—*Edinburg Medical Journal.*

**Antipyrin in Disordered Stomach.**—Dr. Thos. B. McBride writes to the Philadelphia *Medical Times* as follows: Some time since I was called to see a patient suffering from an excessively sensitive stomach. Nothing would be retained. This condition had lasted two days when I saw the case. Everything, almost, had been used, with no avail. I found the patient exhausted, and a temperature of 102½°. Knowing quinine would be vomited, I ordered 3-grain doses of antipyrin, with a view to lowering the temperature. To my satisfaction, the drug was retained by the stomach, and seemed to have a sedative effect on that organ, as there was no vomiting after the first dose.

This called my attention to the drug as a remedy for obstinate vomiting, and I used it in the following cases with beneficial results:

No. 2—Young woman, twenty-four, vomiting from pregnancy. This trouble often baffles all medication. Antipyrin in 4-grain doses to be taken each morning before rising was my treatment, and after the second day there was no further trouble, except when the medicine was not taken, when the vomiting would return.

Nos. 3 and 4—Two children, thirteen months and two years, both suffering from disordered stomach incident to teething, which were both controlled by this drug after other remedies had failed.

I think the above cases warrant me in giving to the profession my experience with the remedy, and would be pleased to hear of the effect produced in the hands of others.

**Early Removal of Sutures.**—In the American Practitioner, October, 1873, page 222, may be found an account of a case of entropium which was subjected to the operation of dividing the free border of the lid from the punctum lachrymarium to the outer canthus, and the removal of a narrow elliptical fold of integument, including the middle portion of the orbiculari muscle as it lay upon the upper lid, quite exposing the tarsal cartilage.

The strip of integument containing the eyelash was slipped up on the surface of the tarsus and secured with interrupted silk sutures.

The operation was done May 6, 1873. Upon the following day, union having occurred between the apposed surfaces, the sutures were removed. Since that time, both in private practice and at the Hospital College clinic, sutures about the eyelids and face have generally been removed in twenty-four hours. This rule is so important, as tending to prevent suppuration along the course of the suture in the wound, and thus secure the most perfect closure of skin-wounds, that surgeons should observe it whenever practicable.—*Progress.*

**Hyperpyrexia in Rheumatic Fever.**—Before the Academy of Medicine in Ireland, Dr. A. N. Montgomery read a paper on the above subject: The patient was a lady, æt. twenty-seven, who, on a passage from Liverpool to Valparaiso, was attacked on board ship with all the symptoms of acute articular rheumatism. The temperature rose on the third day to 102°. She was put on the salicylate of sodium treatment, and in ten days the temperature was normal, and the patient was walking about free from pain and fever. However, after a convalescence of ten days she got wet through while in bed, by a sea coming through at a port-hole, and all her old symptoms returned, with a rapid rise of temperature to 106° within thirty-six hours. A full dose of 20 grains of quinine failing to have any effect in lowering the temperature—which continued to rise rapidly until the thermometer showed 108°—as a *dernier resort*, resources were then had to the ice pack, using towels wrung out of iced water, the application being gradually extended from the head to the lower extremities; and the patient being then rolled up in blankets, she was kept thus for ten minutes at a time. After the third and last application the temperature had fallen to 97.8°, thus making a sudden fall of 10.2° in the space of forty minutes, with a marked relief to the head symptoms, as she completely lost her delirium, spoke rationally, and expressed her delight at feeling the icy cloths around. The temperature, after three days, rose again to 102°, but never went higher; and from this point the pains diminished, and she finally landed in Chili thirty days after the first attack, free from any acute rheumatic symptoms other than a damaged mitral valve. The author remarked that though this plan of treatment was not free from danger from endocarditis, considering the weakened condition of the heart, still that in this case, when other methods had failed to reduce the high temperature, it had succeeded, when the case if let alone must have ended fatally. He, therefore, thought that he was justified in using it.—*Quarterly Compendium.*

**The Kawa Plant.**—In the Berlin Medical Society, on the 20th of January, Dr. H. Lewin made a report of some experiments with the kawa plant. The active principle of the drug does not contain nitrogen, but belongs to the resin series of coloring matters. A short time previously Dr. L. had had an opportunity of witnessing some effects of the drug in the case of a colleague who was making some experiments with it on his own person.

When six or seven light scratches were made on the skin, and the part sprayed with a solution of the active principle, a slight diminution of sensibility occurred. Having had his curiosity aroused by this, Dr. L. made further experiments, and ascertained that if a needle is introduced beneath the skin into the cellular tissue, after the injection of this substance, it may be thrust around in any direction without eliciting the least sensibility. Dr. L. then made some experiments on himself, when he observed that in the entire region of the injection, as far as the resin green extended, from Sunday till Thursday noon there was no sensibility to touch, and only the slightest pricking sensation resulted from the employment of a strong induction current. When the substance has been applied to the tongue, quinine may be taken into the mouth without the least sense of bitter being experienced.—*Ex.*

**Cubeb-Inhalations in Diphtheria.**—Dr. R Couetoux, in the Bulletin Generale de Therapeutique, reports two cases, aged six and nine years, respectively, of diphtheritic croup treated under very unfavorable circumstances, in a cottage with a dung-hill under the window, and containing only one 15-feet square room, in which the family lived. When death was apparently imminent, and tracheotomy had been refused as a last resort, he ordered inhalations of steam, tinctured with cubebs, of which 25 grammes were placed in the boiler at once, the steam being conveyed by tubes to the beds of the children to be inhaled by them. Both made a good recovery. Dr. Couetoux considers that cubebs is capable of being very easily employed in the form of vapors, and that, used in this way against diphtheria, it is more powerful than eucalyptus, glycerine, tar, or the essence of terebenthine. However, he does not urge it to the exclusion of other remedies. In cases where it seems to be too irritating, the addition of glycerin might be advisable. The use of tonics, and rectal feeding when the stomach was rebelious, were also parts of the treatment.—*Phil. Med. Times.*

**Pelvic Anodyne.**—As an example of pelvic anodyne with special reference to the ovaries. Dr. Alfred Meadows (*British Medical Journal*) knows of none that can compare with conium, or, better still, with the alkaloid conia, used in the form of a vaginal pessary. In all cases, whether neuralgic or inflammatory, in which the ovaries are the seat of pain, conia is quite a specific. No drug that Dr. Meadows knows of acts with equal certainty and success. Lastly, he supposes we are all agreed that bromide of potassium exercises a most powerful influence upon the ovaries. No drug, in his experience, can equal it in controlling ovarian mucorrhagia; it not only limits the flow in these particular cases, but it seems also to exercise a distinct and controlling influence upon ovarian mucorrhagia, so far as regards its too frequent menstruation, or, as he prefers to say, too frequent ovulation.—*Weekly Medical Review.*

**Total Extirpation of the Tongue by Kocher's Method.**—A case of true epithelioma involving the right half of the tongue opposite the last molar teeth. The whole tongue having been found to be involved, it was drawn out and removed at the level of the hyoid bone after the side of the mouth had been opened by incision under the jaw. The patient was discharged cured twenty-eight days after the operation. The operator considered the thorough carrying out of aseptic treatment a very important factor in securing this rapid result, and his modification of the

"sticky" gauze of Billroth greatly facilitated it. His formula is: Resin, castor oil, alcohol, and iodoform in the proportion of 10, 6, 15, and 5 parts respectively, mixed together and applied to the gauze.—*Proceedings New York Surgical Society.*

**Treatment of Severe Cases of Burning by Hebra's Water-bed.**—In the Glasgow *Medical Journal*, Dr. W. T. Laurie has a short article calling attention to the value of Hebra's water-bed in the treatment of severe burns and other extensive skin wounds from whatever source, and pointing out the advantages it possesses over the ordinary treatment by dressings. Hebra's water-bed, according to the description quoted from Kaposi, consists of an iron framework in the form of a bed suspended by chains in a zinc trough, and carrying a matress on which the patient is laid. When in use, the trough is filled with warm water, and the patient is gently lowered into it, so as to be entirely immersed, exceping, of course, the head. The temperature of the water is then adjusted to his comfort, and it seems the best temperature is about 104° F. He is kept constantly in the bath, leaving it for functional purposes only, till the wounds have sufficiently healed to allow him to leave it for good.

The advantages claimed for the water-bed are:

1. That it abolishes pain and the consequent need for opium.

2. It does away with the torture and danger of repeated dressings.

3. It provides perfect facility for the removal of discharges, and is absolutely aseptic.—*Medical and Surgical Repoter.*

**Morphine Subcutaneously in Infantile Convulsions.**—Dr. C, S. Schofield, of Boston, reports the case of a child eighteen months of age, previously healthy, whom he had been called to see on account of eclampsia. The child had been in convulsions for two hours, and had been given emetics, hot baths, and mustard to the feet, without any benefit. The writer at once administered $\frac{1}{8}$ grain of sulphate of morphine hypodermically, which was repeated at the end of twenty minutes—no effect having been produced by the first dose. This was also followed by no improvement, and a third injection was administered twenty minutes later. This was effectual in controlling the convulsions, and by the expiration of an hour from the time of administration of the first dose the child was sleeping quietly. When seen the following morning the child had taken food as usual, and was apparently as well as ever.—*Medical Record.*

**Death from Anæsthetics in 1885.**—The deaths during narcosis in the past year in England have been collated by Dr. E. H. Jacobs, and reported in the British Med. Journal. Twelve deaths occurred in chloroform narcosis during an unusually severe list of operations, only four being trivial. The casualties from ether are but three, a small record considering the general use of ether in English surgical centers. No death is recorded from methylene bichloride, or from mixtures of ether and chloroform.—*Cal. Prac.*

**After Treatment of Cataract.**—At the St. Louis meeting of the American Medical Association, Dr. Michael advocated the plan of using a light bandange to the eyes after cataract operations and iridectomies, and allowing the patients to be in a lighted room, where friends can come and read to them. Dr. Michael's plan was not favorably received at St. Louis, but it has been tried by Dr. Chisolm, of Baltimore, who reports fourteen cataracts and four iridectomies treated in this way. After the removal of a cataract or the performance of an iridectomy, the eyes if a cataract, the eye if an iridectomy, is closed in its normal position, and a bit of isinglass plaster, about two and a half inches long by one inch wide, is then rendered flaccid by immersion in some germicide fluid, and is neatly applied to the surface of the closed lids. When dried this forms a close, firm band. The patient is then allowed the full liberty of his room, and is not shut up in darkness, as was formerly deemed essential.—*Scientific American.*

**Convulsions During Pregnancy Treated Successfully by Subcutaneous Injections of Pilocarpin.**—In the London *Lancet* for April 3, 1886, Dr. P. Horrocks reports a case of convulsions during pregnancy successfully treated by subcutaneous injections of pilocarpine. This was a second case within a year. The patient's urine contained about one-third albumen with no casts; she had convulsions and had been unconscious for some time. One-third of a grain of a fresh solution of pilocarpin was injected subcutaneously. In both cases there was complete relief of the symptoms, rapid dilatation of the os, and quick expulsion of the child after the administration of the drug. It would appear that pilocarpine must be looked upon as an ecbolic. In both cases the children died. Whether this was owing to the pilocarpine, or the convulsions, or the uterine constructions set up by pilocarpine, or some other cause, must be left to future investigations.—*N. O. Medical Journal.*

**Ergot for Dysentery.**—In a case of a man, forty-four years of age, suffering with acute dysentery (Le Progres Medical), in which bismuth and laudanum had failed, Dr. Du Rocher gave calomel in large doses with no better results. He then resorted to powdered ergot, of which 45 grains were given in six doses through the night. The next day there were only two evacuations of the bowels, without a trace of blood. The ergot was continued, and twenty-four hours later the loose discharges were entirely suppressed, and convalescence commenced. The reporter was favorably impressed with the results from the remedy.—*Phil. Med. Times.*

**The Editor's Work.**—Some people estimate the ability of a periodical and the talent of its editors by the quantity of its original matter. It is comparatively an easy task for a frothy writer to string out a column of words upon any and all subjects. His ideas may flow in one weak, washy, everlasting flood, and his command of the language may enable him to string them together like bunches of onions, and yet his paper may be but a meager and poor concern. Indeed, the mere writing part of editing a paper is but a small portion of the work. The care, the time employed in selecting is more important, and the fact of a good editor is better shown by his selections than anything else, and that, we know, is half the battle. But we have said, an editor ought to be estimated, his labor understood and appreciated by the general conduct of his paper—its tone, its uniform. consistent course, aims, manliness, dignity, and its propriety.—*Courier-Journal.*

**Treatment of Facial Neuralgia by Cocaine.**—Dr. D. Coninck, of Ledeberg-les-Gant, writes to the *Scalpel*, of Liege, that the effects of hydrochlorate of cocaine in facial neuralgia and in cephalalgia having its seat in the temporal region are surprising. The pain, be it ever so intense, will instantaneously cease on applying to the auditory canal one minim of a solution of 1 per cent. of this salt, by means of a camel-hair brush. This signal effect, however, will only continue for a few hours, after which a repeated application may be required. Hydrochlorate of cocaine has never failed in the many cases of these kinds of neuralgia, treated in that manner by Dr. de Coninck. In neuralgia of the fifth nerve and its branches, however, the results were less certain and less satisfactory, owing, perhaps, to the superficial mode of its employment.—*Medical and Surgical Reporter.*

**Fluorides of Ammonia and Iron.**—Dr. John Lucas, of Bombay, has employed the fluoride of ammonia in the treatment of hypertrophy of the spleen. The drug appears to have antipyretic and antiperiodic properties, as proved in cases of ague. Nausea was produced at first, but afterwards large doses could be borne. The appetite after a time improves under its use. By giving the drug after meals, its nauseant and purgative actions are greatly lessened. It certainly appears to possess the merit of excelling any other method of treatment of hypertrophied spleen with which we are acquainted. Dr. Lucas began with 5 minim doses, but in future would be disposed to give 20 or 30 minims well diluted. The fluoride of iron would perhaps be preferable to the ammonia salt, on account of its hæmatinic properties.—*Lancet.*

**Digitalis Treatment of Pneumonia.**—Petrescu, of the Military Hospital at Bucharest, reports remarkable success in the treatment of acute pneumonia from the administration of large doses of digitalis, given in the form of recent infusion. His conclusions, based upon observation of three hundred and fifty cases, are as follows :

1. Digitalis produces antiphlogistic and direct effects only when given in appropriate dose.

2. This dose is from 4 to 6 grammes (ʒ j–jss) given during the twenty-four hours, and continued for several days. In this way he has given as much as 20 grammes (ʒ v) inside of three days.

3. The treatment of pneumonia by digitalis is the only method of reducing the mortality of the disease to a minimum.—*Philadelphia Med. Times.*

